# Gingival Squamous Cell Carcinoma: Diagnostic Challenges and Clinical Implications

**DOI:** 10.1002/ccr3.72664

**Published:** 2026-05-05

**Authors:** Gitanjali Subedi, Arjun Hari Rijal, Simant Lamichhane, Pratibha Poudel, Sachita Thapa, Manoj Humagain

**Affiliations:** ^1^ Department of Periodontology and Oral Implantology Kathmandu University School of Medical Sciences, Dhulikhel Hospital Dhulikhel Nepal; ^2^ Department of Oral Pathology Kathmandu University School of Medical Sciences, Dhulikhel Hospital Dhulikhel Nepal

**Keywords:** gingival carcinoma, malignant neoplasms, misdiagnosis, selective neck dissection

## Abstract

Oral squamous cell carcinoma (OSCC) is one of the highly invasive malignancies globally, accounting for the vast majority of all oral cancers, that is, more than 90%. Carcinomas of the gingiva represent a distinct subgroup of OSCC, constituting approximately 12% of all OSCC cases. These neoplasms might mimic the inflammatory and reactive state of the periodontium, often leading to a delay in diagnosis and hence, poorer prognosis. A case of gingival squamous cell carcinoma in a 48‐year‐old female patient has been reported here, in which the patient had no risk factors for the development of this neoplasm. The patient presented with a 3‐month history of pain and burning sensation in the lower left back region of the jaw. Clinical examination showed a proliferative lesion extending from the marginal gingiva to the buccal vestibule adjacent to teeth 36 and 37. Given the clinical manifestations and differential diagnosis of infectious granulomatous lesions and malignant neoplasms, an excisional biopsy was performed and a diagnosis of well‐differentiated squamous cell carcinoma was made. The patient was subsequently referred to the Oral and Maxillofacial Surgery Department for further treatment, which included surgical excision of the tumor with segmental mandibulectomy and selective neck dissection. The patient is still being monitored one and a half years after the treatment and does not show any signs of recurrence. Therefore, the lesion must be identified early to start treatment, stop metastases, and enhance the prognosis.

## Key Clinical Message

The similarities of gingival squamous cell carcinoma with common periodontal lesions could cause a delay in diagnosis or even a misdiagnosis. In cases with suspected gingival lesions, differential diagnosis of squamous cell carcinoma should always be considered because it can have a favorable prognosis if the treatment is initiated early.

## 1 Introduction

As the most common malignant neoplasm affecting the structures of the oral cavity, squamous cell carcinoma (SCC) accounts for over 90% of all oral malignant lesions [1]. The lateral/ventral aspect of the tongue and floor of the mouth are considered the most common sites for intraoral SCC, excluding the carcinoma of the lip vermilion (which has a different etiology) [2]. Gingival lesions comprise approximately 12% of all intraoral cases [1]. Compared to the maxilla, the mandible has a higher likelihood of having gingival SCC, most of which are located in the molar region [3]. However, gingival carcinoma is frequently diagnosed too late or often misdiagnosed because it resembles common benign inflammatory lesions. Early‐stage carcinoma can mimic an erosion, small ulcer, exophytic mass, or a periodontal lesion. They can also present as an asymptomatic erythematous or white lesion, or both [4]. So, when treating gingival lesions, especially in elderly patients, the SCC of the gingiva should be taken into account when making a differential diagnosis. To begin treatment early and improve the prognosis, it is imperative to diagnose gingival lesions as soon as possible.

Tobacco abuse, either in smoked or smokeless form, alcohol consumption, and betel nut chewing can be regarded as significant contributing factors to SCC, even though the exact etiology of this condition is unknown [4]. The tumor has a strong tendency to invade nearby osseous structures, especially the maxilla and alveolar bone, and a large proportion of cases exhibit bone involvement at the time of diagnosis [5]. Major complications include mandibular/maxillary bone invasion, tooth loss, fistula formation, and trismus. The aggressive growth of the lesions can quickly penetrate and harm nearby structures, including the airway, resulting in serious infections, bleeding, and obstruction of the airway [6]. Depending on the location, stage, and overall health of the patient, treatment for gingival SCC is often multidisciplinary and involves surgery, radiation therapy, and systemic therapy. First line of therapy for early stages is surgical excision. However, radiation therapy alone or in conjunction with chemotherapy is also considered as the main therapeutic strategy for gingival SCC [7].

Here, we document a case of a 48‐year‐old female patient with gingival squamous cell carcinoma on the left mandibular posterior area.

## 2 Case History and Examination

A 48‐year‐old female patient was presented to the Periodontics Department, with the chief complaint of pain and burning sensation on the lower left posterior region of the jaw for 3 months. The pain was spontaneous, continuous, severe in intensity, non‐radiating type, aggravated on taking hot and spicy foods and touching while relieved on taking analgesics. Medical history was non‐significant. There was no history of alcohol and tobacco abuse, both in smoked and smokeless form, and no positive family history of malignancy. According to the patient, she had visited a private dental clinic for the same problem, as soon as she started developing the clinical symptoms that is, 3 months before reporting to our department. She mentioned that the sole intervention she received was oral prophylaxis followed by two courses of antibiotics (a combination of Amoxicillin and Metronidazole), as prescribed by a private dental practitioner. Since there was no improvement in the clinical symptoms, the patient was referred to our department.

Upon extra‐oral examination, a single, non‐tender, mobile, and firm submandibular lymph node was palpated on the left side. Intra‐oral examination revealed an ulcer‐proliferative lesion extending from the marginal gingiva to the buccal vestibule, measuring approximately 15 × 8 mm adjacent to teeth 36 and 37, with irregular necrotic margins (Figure 1). Uniformly white plaques were also evident, seen on the underlying vestibular fundus, as well as on the mesial attached gingiva adjacent to tooth 35. Intra‐oral Periapical Radiograph (IOPAR) of the region revealed bone loss on the furcation area in relation to 36, while the interdental bone on both sides was intact (Figure 2). Complete blood investigations were done, which were within the normal range.

**FIGURE 1:**
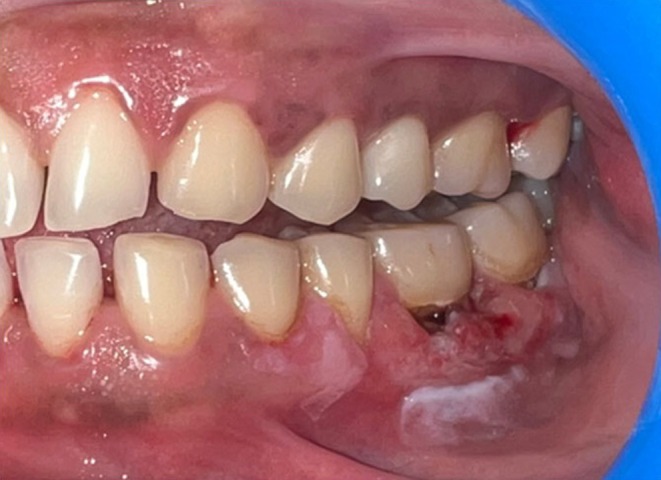
An ulcerative lesion extending from the marginal gingiva to the buccal vestibule, adjacent to teeth 36 and 37.

**FIGURE 2:**
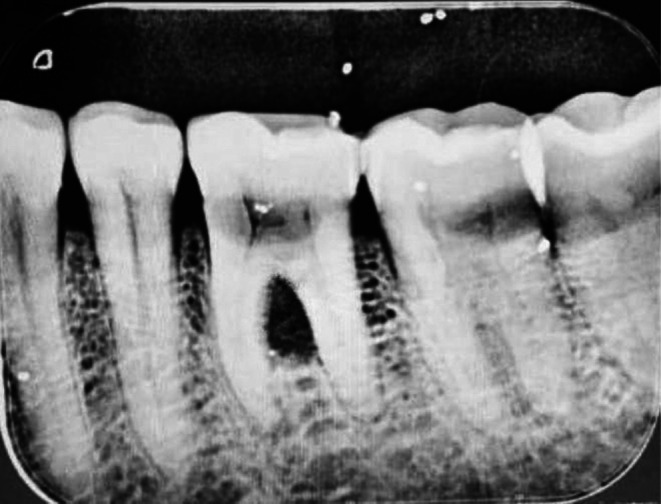
IOPAR of the area showing bone loss on the furcation area w.r.t 36, while the interdental areas being intact.

## 3 Differential Diagnosis, Investigations and Treatment

In view of the clinical manifestations, we made a differential diagnosis which included SCC, verrucous carcinoma, homogenous leukoplakia as well as Necrotizing Ulcerative Periodontitis (NUP). However, because of the lesion's persistence and poor responsiveness to medical therapy, NUP was excluded from the differential diagnosis and a strong suspicion of non‐infectious and non‐inflammatory disease was considered. Under local anesthesia, every visible part of the lesion was removed and sent for histopathological analysis (Figures 3 and 4).

**FIGURE 3:**
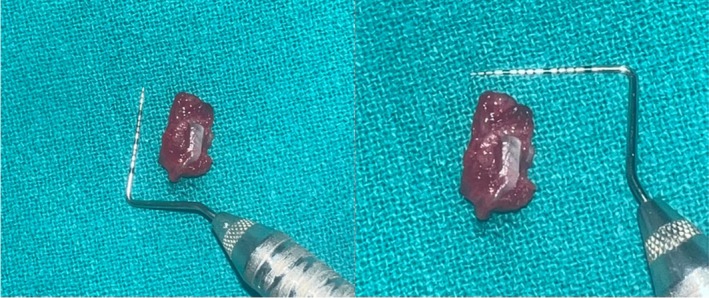
Excised gingival tissue mass, measurements done with UNC‐15 probe.

**FIGURE 4:**
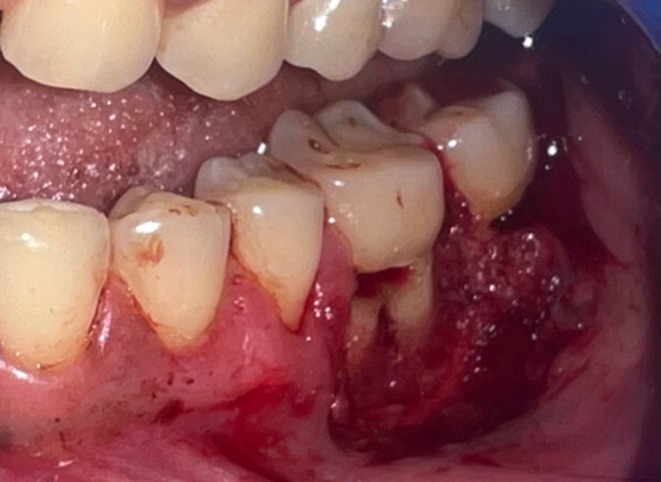
The area showing loss of alveolar bone on the buccal aspect after excision of the mass.

Histopathological examination revealed dysplastic keratinized stratified squamous epithelium overlying the fibro‐cellular connective tissue stroma. The underlying stroma was infiltrated with tumor cells forming islands and sheets. The tumor cells showed dysplastic features such as increased cytoplasmic ratio, cellular and nuclear pleomorphism, anisocytosis, anisonucleosis, atypical mitotic figures, and abundant keratin pearl formation (Figure 5). Intense inflammatory cell infiltration comprising chiefly of lymphocytes and plasma cells was also noted. These microscopic features led to the final diagnosis of well‐differentiated squamous cell carcinoma (TNM staging: T_1_N_1_M_0_).

**FIGURE 5:**
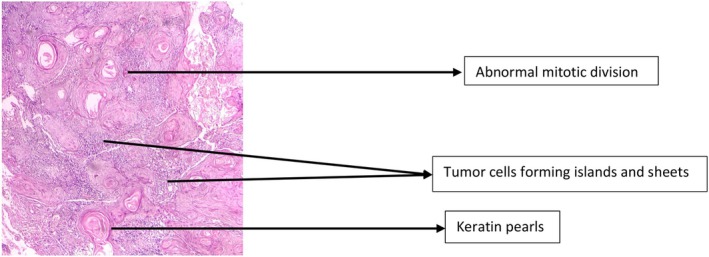
Histopathological examination showing dysplastic keratinized stratified squamous epithelium overlying the fibro‐cellular connective tissue stroma.

T (Size of the primary tumor) – T_1_, because the size of the primary tumor was < 2 cm.

N (Regional lymph node involvement) – N_1_, because it was positive for lymph node level IB metastasis.

M (Distant metastasis) – M_0_, because of no distant metastasis.

The patient was then referred to the Department of Oral and Maxillofacial Surgery for further treatment, where a wide local excision of the lesion with segmental mandibulectomy and selective neck dissection (lymph node level I–IV), followed by removal of regional lymph nodes was done under general anesthesia. The patient was then referred to Kathmandu Cancer Hospital for radiotherapy, 5 daily fractions a week fractionated for 7 weeks, each session of 50 Gy.

## 4 Outcomes and Follow up

The patient is still being monitored, one and half years after the end of the treatment and she is not exhibiting any signs of recurrence at present. However, the patient presented with the signs of radiation mucositis (Figure 6).

**FIGURE 6:**
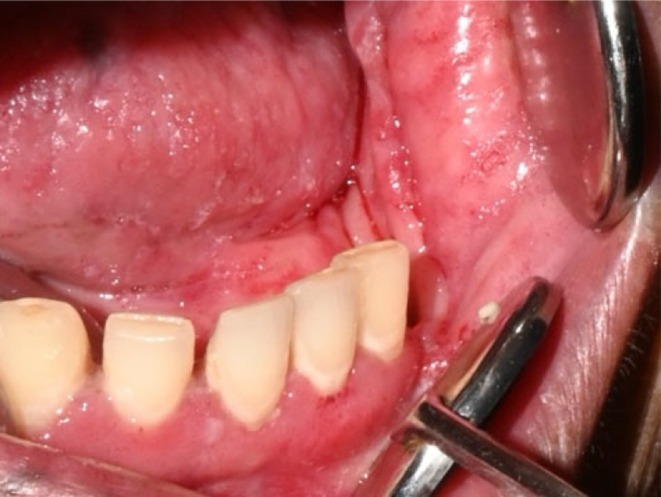
Post‐operative image after 1.5 years of segmental mandibulectomy.

## 5 Discussion

According to the estimates from the GLOBOCAN project in 2018, there are at least 354,864 new cases of oral cancer (mostly SCC) worldwide each year, that is, 2.0% of all malignancies [8]. It is approximated that carcinoma of the oral cavity in conjunction with lip cancer ranks sixth among all cancers in Nepal and fourth among cancers in men [9]. Gingival carcinoma represents a rare but distinct subgroup of OSCC. Carcinoma of the gingiva is an insidious disease that often lacks the clinical manifestation of the malignant tumor and hence is misdiagnosed as other inflammatory conditions of the periodontium, subsequently influencing the patient's prognosis and chance for survival. Usually originating from keratinized mucosa in a posterior location, it frequently destroys the underlying bone, resulting in tooth mobility [6].

Tobacco abuse, either in smoked or smokeless form, alcohol consumption, and betel nut chewing can be regarded as significant contributing factors to SCC, even though the exact etiology of this condition is unknown [4]. On the other hand, the incidence of oral cancer among young people has been rising over time, despite a decline in smoking and alcohol consumption [10]. Thus, it has been reported that the association between these factors and carcinogenesis may not be as strong as thought. In contrast, Jiang et al. [11] have documented a strong correlation between smoking and alcohol intake and gingival SCC. The majority of case studies [3] have documented a predilection of gingival SCC for mandibular arches; however, Lubek et al. discovered nearly equal amounts of gingival lesions in both arches [12]. The average age of onset of gingival SCC is similar to that of other intra‐oral SCC. Historically, it was considered as the disease of the elderly, but there has been a recent shift in the prevalence of these lesions in younger patients who do not meet the established risk factors [13]. Since isolated cases of gingival SCC in pediatric patients have also been reported, clinicians should not discount the possibility of gingival SCC in this patient population [14].

The diagnosis of gingival SCC is quite difficult due to its resemblance to other periodontal inflammatory conditions as well as to other pre‐malignant conditions. In the present case, uniform white plaques seen on the vestibular area of the underlying tooth may be suggestive of homogenous leukoplakia, which has a very low malignant transformation rate. Brooks et al., in a case report, described a case of SCC of the gingiva in a 60‐year‐old female patient, with an unexpected clinical presentation. The patient displayed persistent gingival bleeding with a moderately inflamed and ulcerated border on the maxillary right molar region. She had previously experienced right‐sided stage 2 breast cancer. An incisional biopsy was carried out after taking into account her medical history and the possibility of metastases. According to the histopathological analysis, the lesion was identified as a moderate to well‐differentiated gingival SCC [4]. Another similar case was reported by Khan et al., where the clinical presentation of the patient was misleading, giving the diagnosis of Necrotizing Ulcerative Periodontitis (NUP), yet the diagnosis of well‐differentiated SCC was confirmed by the histopathological results [15].

In cases of refractory chronic periodontal diseases, the clinician must be skeptical of any changes in the morphology of the gingiva, for instance, desquamations, ulcerations, papillae loss, and verrucous and hyperplastic growths, as these may be early clinical indicators of the upcoming malignancy. If suspicious gingival lesions remain after the etiological agents have been removed for longer than 2 weeks, a thorough history, clinical examination, and biopsy should be carried out for histopathological analysis in order to rule out malignancy and improve success and survival rates of the treatment.

Therefore, when treating rare periodontal disorders, gingival SCC should always be taken into account as a differential diagnosis. In situations like these, periodontists can be lifesavers because they can help rule out this aggressive disease even in the absence of risk factors.

## Author Contributions

**Gitanjali Subedi:** conceptualization, data curation, methodology, writing – original draft. **Arjun Hari Rijal:** validation, writing – review and editing. **Simant Lamichhane:** writing – review and editing. **Pratibha Poudel:** investigation. **Sachita Thapa:** resources. **Manoj Humagain:** supervision.

## Funding

This case report is self‐funded.

## Ethics Statement

All procedures performed in the study involving human participants were in accordance with the ethical standards of the institutional and/or national research committee and with the 1964 Helsinki Declaration and its later amendments or comparable ethical standards.

## Consent

Verbal as well as written consent was obtained from the patient before performing the procedure. Consent to publish this information was also obtained from the patient.

## Conflicts of Interest

The authors declare no conflicts of interest.

## Data Availability

Data supporting this research article are available from the corresponding author or first author on reasonable request.
